# *N*-{4-[(3,4-Dimethyl­phen­yl)(eth­yl)sulfamo­yl]phen­yl}-*N*-ethyl­acetamide

**DOI:** 10.1107/S1600536810045708

**Published:** 2010-11-13

**Authors:** Peter John, Saima Khizar, Islam Ullah Khan, Shahzad Sharif, Edward R. T. Tiekink

**Affiliations:** a Materials Chemistry Laboratory, Department of Chemistry, Government College, University, Lahore 54000, Pakistan; b Department of Chemistry, University of Malaya, 50603 Kuala Lumpur, Malaysia

## Abstract

When viewed down the central S⋯N axis of the title compound, C_20_H_26_N_2_O_3_S, it is apparent that the mol­ecule adopts a *gauche* conformation with all O atoms lying to one side of the central benzene ring; the carbonyl O atom is directed away from the central ring and the *N*-bound ethyl groups lie to one side of the mol­ecule. Supra­molecular helical chains aligned along the *b* axis and sustained by C—H⋯O contacts feature in the crystal packing. These are consolidated in the three-dimensional structure by C—H⋯π inter­actions.

## Related literature

For background to the pharmacological uses of sulfonamides, see: Korolkovas (1988 ▶); Mandell & Sande (1992 ▶). For related structures, see: Sharif *et al.* (2010 ▶); Khan *et al.* (2010 ▶).
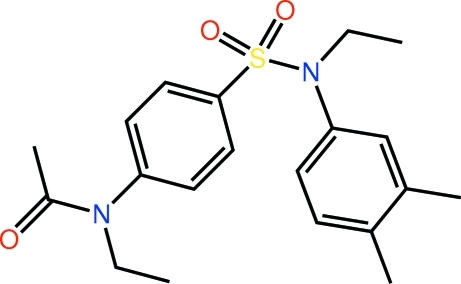


## Experimental

### 

#### Crystal data

C_20_H_26_N_2_O_3_S
*M* *_r_* = 374.51
Monoclinic, 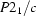

*a* = 8.0882 (2) Å
*b* = 11.5978 (3) Å
*c* = 21.2717 (5) Å
β = 97.194 (1)°
*V* = 1979.69 (8) Å^3^
*Z* = 4
Mo *K*α radiation
μ = 0.19 mm^−1^
*T* = 293 K
0.28 × 0.14 × 0.08 mm

#### Data collection

Bruker APEXII CCD diffractometer
Absorption correction: multi-scan (*SADABS*; Sheldrick, 1996 ▶) *T*_min_ = 0.692, *T*_max_ = 0.895
16612 measured reflections
4079 independent reflections
3325 reflections with *I* > 2σ(*I*)
*R*_int_ = 0.029

#### Refinement

*R*[*F* ^2^ > 2σ(*F* ^2^)] = 0.039
*wR*(*F* ^2^) = 0.122
*S* = 1.01
4079 reflections
240 parameters
H-atom parameters constrained
Δρ_max_ = 0.24 e Å^−3^
Δρ_min_ = −0.26 e Å^−3^

### 

Data collection: *APEX2* (Bruker, 2007 ▶); cell refinement: *SAINT* (Bruker, 2007 ▶); data reduction: *SAINT*; program(s) used to solve structure: *SHELXS97* (Sheldrick, 2008 ▶); program(s) used to refine structure: *SHELXL97* (Sheldrick, 2008 ▶); molecular graphics: *ORTEP-3* (Farrugia, 1997 ▶) and *DIAMOND* (Brandenburg, 2006 ▶); software used to prepare material for publication: *publCIF* (Westrip, 2010 ▶).

## Supplementary Material

Crystal structure: contains datablocks global, I. DOI: 10.1107/S1600536810045708/hg2743sup1.cif

Structure factors: contains datablocks I. DOI: 10.1107/S1600536810045708/hg2743Isup2.hkl

Additional supplementary materials:  crystallographic information; 3D view; checkCIF report

## Figures and Tables

**Table 1:** Hydrogen-bond geometry (Å, °) *Cg*1 is the centroid of the C3–C8 ring.

*D*—H⋯*A*	*D*—H	H⋯*A*	*D*⋯*A*	*D*—H⋯*A*
C8—H8⋯O1^i^	0.93	2.54	3.455 (2)	170
C10—H10a⋯*Cg*1^ii^	0.96	2.93	3.728 (2)	142

Symmetry codes: (i) 
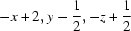
; (ii) 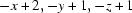.

**Fig. 1.**
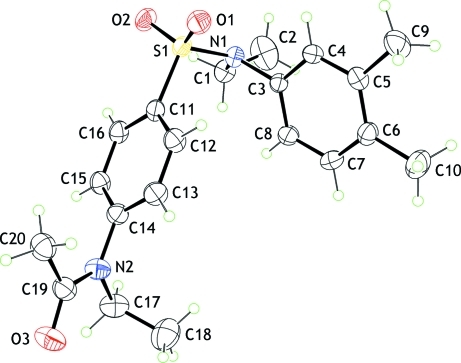
The molecular structure of (I) showing the atom-labelling scheme and displacement ellipsoids at the 35% probability level.

**Fig. 2.**
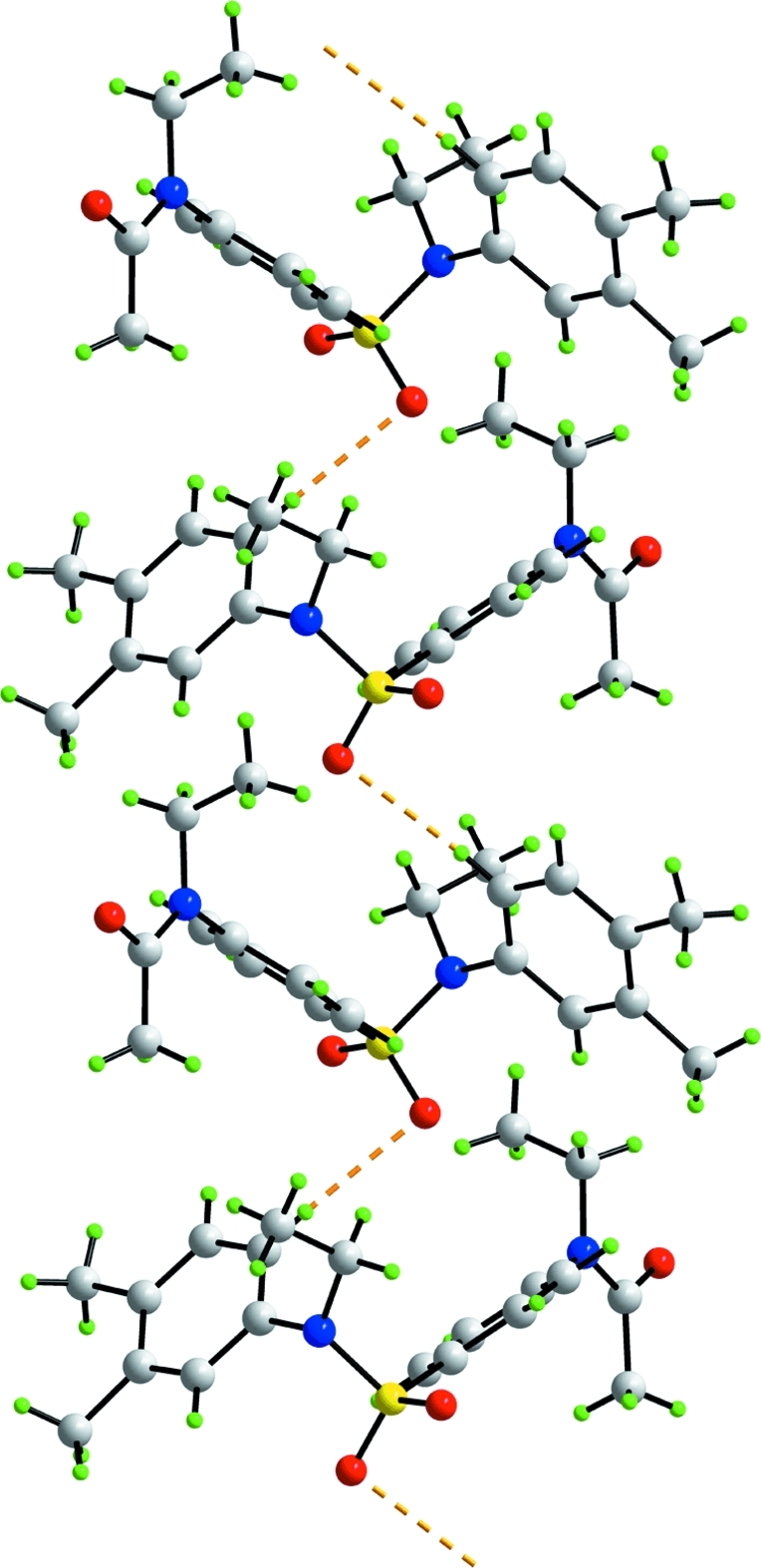
A view of the helical supramolecular chain along the b axis in (I). The C—H···O contacts sustaining this chain are shown as orange dashed lines.

**Fig. 3.**
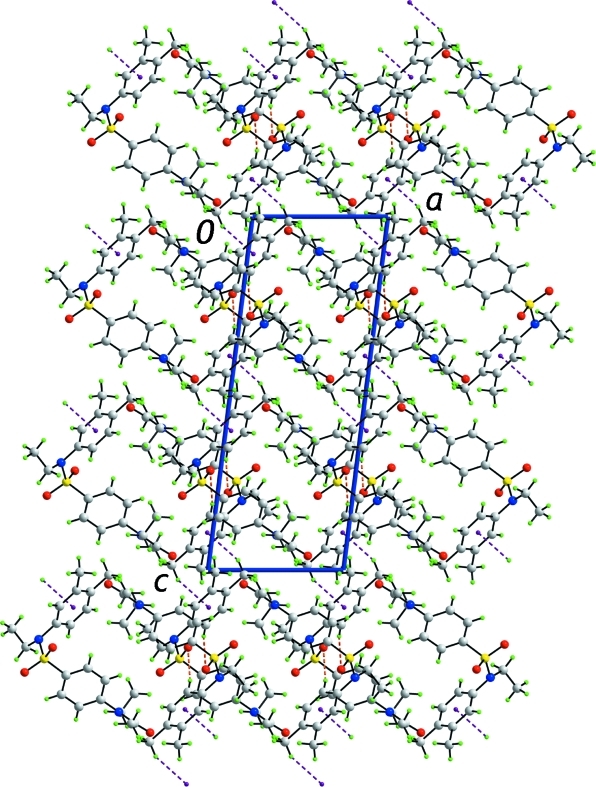
View in projection down the b axis of the unit-cell contents for (I). The C—H···O and C—H···π contacts are shown as orange and purple dashed lines, respectively.

## supplementary crystallographic information

### Comment

In connection with on-going structural studies of sulfonamides (Sharif *et al.*, 2010; Khan *et al.*, 2010), of interest owing to their biological properties (Korolkovas, 1988; Mandell & Sande, 1992), the title compound, (I), was investigated.

With reference to the central benzene ring in (I), Fig. 1, the S1 [deviation = -0.068 (1) Å] and N2 [-0.005 (1) Å] atoms are co-planar. Both sulfonamide-O atoms lie to the same side of the plane as does the carbonyl-O atom, which is directed away from the ring, with the remaining substituents lying to the other side. When viewed down the S1···N2 vector, both N-bound ethyl groups lie to the same side of the molecule. Similarly, when viewed down the S1···N2 vector, the molecule has a *gauche* conformation.

In the crystal packing, molecules are connected into a helical supramolecular chain along the *b* axis *via* C—H···O contacts occurring between benzene-H and sulfonamide-O atoms, Table 1 and Fig. 2. The chains are consolidated in the crystal packing by C—H···π interactions, Table 1 and Fig. 3.

### Experimental

A mixture of *N*-{4-[(3,4-dimethylphenyl)sulfamoyl]phenyl}acetamide 100 mg (0.314 mmol) and sodium hydride 85 mg (0.78 mmol) in *N*,*N*-dimethylformamide (10 ml) was stirred at room temperature for 30 min. followed by addition of ethyl iodide 199 µl (0.785 mmol). Stirring was continued for a further 3 h and the contents were poured over crushed ice. The precipitate that formed was isolated, washed and crystallized from methanol solution by slow evaporation; *M*.pt. 472 K.

### Refinement

The C-bound H atoms were geometrically placed (C–H = 0.93–0.97 Å) and refined as riding with *U_iso_*(H) = 1.2–1.5*U_eq_*(C). In the final refinement four low angle reflections evidently effected by the beam stop were omitted, *i.e*. (100), (002), (011) and (111).

### Crystal data

**Table:**

C_20_H_26_N_2_O_3_S	*F*(000) = 800
*M**_r_* = 374.51	*D*_x_ = 1.257 Mg m^−^^3^
Monoclinic, *P*2_1_/*c*	Mo *K*α radiation, λ = 0.71073 Å
Hall symbol: -P 2ybc	Cell parameters from 7015 reflections
*a* = 8.0882 (2) Å	θ = 3.0–27.8°
*b* = 11.5978 (3) Å	µ = 0.19 mm^−^^1^
*c* = 21.2717 (5) Å	*T* = 293 K
β = 97.194 (1)°	Block, colourless
*V* = 1979.69 (8) Å^3^	0.28 × 0.14 × 0.08 mm
*Z* = 4	

### Data collection

**Table:**

Bruker APEXII CCD diffractometer	4079 independent reflections
Radiation source: fine-focus sealed tube	3325 reflections with *I* > 2σ(*I*)
graphite	*R*_int_ = 0.029
φ and ω scans	θ_max_ = 26.5°, θ_min_ = 2.6°
Absorption correction: multi-scan (*SADABS*; Sheldrick, 1996)	*h* = −9→10
*T*_min_ = 0.692, *T*_max_ = 0.895	*k* = −14→11
16612 measured reflections	*l* = −26→26

### Refinement

**Table:**

Refinement on *F*^2^	Primary atom site location: structure-invariant direct methods
Least-squares matrix: full	Secondary atom site location: difference Fourier map
*R*[*F*^2^ > 2σ(*F*^2^)] = 0.039	Hydrogen site location: inferred from neighbouring sites
*wR*(*F*^2^) = 0.122	H-atom parameters constrained
*S* = 1.01	*w* = 1/[σ^2^(*F*_o_^2^) + (0.0713*P*)^2^ + 0.4585*P*] where *P* = (*F*_o_^2^ + 2*F*_c_^2^)/3
4079 reflections	(Δ/σ)_max_ = 0.001
240 parameters	Δρ_max_ = 0.24 e Å^−^^3^
0 restraints	Δρ_min_ = −0.26 e Å^−^^3^

### Special details

**Table:**

Geometry. All s.u.'s (except the s.u. in the dihedral angle between two l.s. planes) are estimated using the full covariance matrix. The cell s.u.'s are taken into account individually in the estimation of s.u.'s in distances, angles and torsion angles; correlations between s.u.'s in cell parameters are only used when they are defined by crystal symmetry. An approximate (isotropic) treatment of cell s.u.'s is used for estimating s.u.'s involving l.s. planes.
Refinement. Refinement of *F*^2^ against ALL reflections. The weighted *R*-factor *wR* and goodness of fit *S* are based on *F*^2^, conventional *R*-factors *R* are based on *F*, with *F* set to zero for negative *F*^2^. The threshold expression of *F*^2^ > 2σ(*F*^2^) is used only for calculating *R*-factors(gt) *etc*. and is not relevant to the choice of reflections for refinement. *R*-factors based on *F*^2^ are statistically about twice as large as those based on *F*, and *R*- factors based on ALL data will be even larger.

### Fractional atomic coordinates and isotropic or equivalent isotropic displacement parameters (Å^2^)

**Table:**

	*x*	*y*	*z*	*U*_iso_*/*U*_eq_	
S1	1.12740 (5)	0.72075 (4)	0.249229 (18)	0.03937 (14)	
O1	1.07590 (16)	0.82013 (10)	0.28110 (6)	0.0509 (3)	
O2	1.25922 (15)	0.73062 (12)	0.21059 (6)	0.0536 (3)	
O3	0.27644 (17)	0.53664 (14)	0.04014 (7)	0.0694 (4)	
N1	1.18549 (16)	0.62329 (11)	0.30379 (6)	0.0376 (3)	
N2	0.52634 (17)	0.52003 (13)	0.09842 (7)	0.0475 (4)	
C1	1.2726 (2)	0.52088 (16)	0.28259 (8)	0.0476 (4)	
H1A	1.1913	0.4620	0.2683	0.057*	
H1B	1.3306	0.5419	0.2470	0.057*	
C2	1.3933 (3)	0.4738 (2)	0.33407 (12)	0.0874 (8)	
H2A	1.4686	0.5337	0.3505	0.131*	
H2B	1.4551	0.4123	0.3178	0.131*	
H2C	1.3347	0.4450	0.3673	0.131*	
C3	1.06969 (18)	0.60395 (13)	0.34975 (7)	0.0339 (3)	
C4	1.06258 (19)	0.68223 (14)	0.39837 (7)	0.0389 (3)	
H4	1.1336	0.7456	0.4015	0.047*	
C5	0.9517 (2)	0.66828 (15)	0.44270 (7)	0.0422 (4)	
C6	0.8482 (2)	0.57149 (16)	0.43848 (8)	0.0442 (4)	
C7	0.8584 (2)	0.49348 (15)	0.39000 (8)	0.0488 (4)	
H7	0.7902	0.4286	0.3873	0.059*	
C8	0.9669 (2)	0.50905 (14)	0.34545 (8)	0.0440 (4)	
H8	0.9704	0.4559	0.3129	0.053*	
C9	0.9443 (3)	0.7571 (2)	0.49396 (9)	0.0631 (5)	
H9A	1.0265	0.8155	0.4903	0.095*	
H9B	0.9660	0.7207	0.5347	0.095*	
H9C	0.8355	0.7916	0.4896	0.095*	
C10	0.7272 (3)	0.5500 (2)	0.48570 (10)	0.0673 (6)	
H10A	0.7868	0.5468	0.5276	0.101*	
H10B	0.6708	0.4781	0.4761	0.101*	
H10C	0.6471	0.6114	0.4834	0.101*	
C11	0.95077 (19)	0.66426 (14)	0.20256 (7)	0.0377 (3)	
C12	0.7959 (2)	0.68032 (16)	0.22264 (8)	0.0454 (4)	
H12	0.7859	0.7231	0.2589	0.055*	
C13	0.6567 (2)	0.63238 (16)	0.18843 (8)	0.0477 (4)	
H13	0.5526	0.6421	0.2020	0.057*	
C14	0.67146 (19)	0.56991 (14)	0.13400 (8)	0.0410 (4)	
C15	0.8263 (2)	0.55379 (15)	0.11401 (8)	0.0435 (4)	
H15	0.8359	0.5115	0.0775	0.052*	
C16	0.96666 (19)	0.60077 (15)	0.14841 (7)	0.0422 (4)	
H16	1.0710	0.5898	0.1353	0.051*	
C17	0.5128 (2)	0.39364 (17)	0.09782 (10)	0.0592 (5)	
H17A	0.6237	0.3608	0.1001	0.071*	
H17B	0.4510	0.3697	0.0579	0.071*	
C18	0.4287 (4)	0.3465 (2)	0.15098 (12)	0.0839 (7)	
H18A	0.4904	0.3684	0.1907	0.126*	
H18B	0.4241	0.2639	0.1480	0.126*	
H18C	0.3177	0.3768	0.1484	0.126*	
C19	0.4017 (2)	0.58325 (17)	0.06710 (8)	0.0485 (4)	
C20	0.4223 (3)	0.71197 (17)	0.06548 (9)	0.0564 (5)	
H20A	0.3588	0.7423	0.0280	0.085*	
H20B	0.5379	0.7305	0.0652	0.085*	
H20C	0.3834	0.7454	0.1022	0.085*	

### Atomic displacement parameters (Å^2^)

**Table:**

	*U*^11^	*U*^22^	*U*^33^	*U*^12^	*U*^13^	*U*^23^
S1	0.0372 (2)	0.0426 (3)	0.0387 (2)	−0.00274 (16)	0.00612 (16)	0.00737 (16)
O1	0.0619 (8)	0.0375 (6)	0.0527 (7)	0.0004 (6)	0.0049 (6)	0.0032 (5)
O2	0.0408 (6)	0.0717 (9)	0.0498 (7)	−0.0092 (6)	0.0114 (5)	0.0127 (6)
O3	0.0518 (8)	0.0747 (10)	0.0762 (9)	−0.0066 (7)	−0.0140 (7)	−0.0079 (8)
N1	0.0376 (7)	0.0394 (7)	0.0365 (6)	0.0037 (5)	0.0071 (5)	0.0036 (5)
N2	0.0397 (7)	0.0436 (8)	0.0584 (9)	0.0001 (6)	0.0028 (6)	−0.0044 (7)
C1	0.0457 (9)	0.0479 (10)	0.0514 (9)	0.0085 (8)	0.0142 (8)	0.0004 (8)
C2	0.0891 (17)	0.0869 (18)	0.0821 (16)	0.0473 (14)	−0.0048 (13)	0.0050 (14)
C3	0.0342 (7)	0.0354 (8)	0.0323 (7)	−0.0006 (6)	0.0043 (6)	0.0021 (6)
C4	0.0388 (8)	0.0380 (8)	0.0392 (8)	−0.0052 (7)	0.0018 (6)	−0.0018 (6)
C5	0.0413 (8)	0.0477 (9)	0.0367 (8)	0.0048 (7)	0.0021 (7)	−0.0035 (7)
C6	0.0393 (8)	0.0528 (10)	0.0416 (8)	0.0021 (7)	0.0086 (7)	0.0060 (7)
C7	0.0494 (10)	0.0434 (9)	0.0544 (10)	−0.0142 (8)	0.0095 (8)	0.0013 (8)
C8	0.0530 (10)	0.0379 (9)	0.0416 (8)	−0.0073 (7)	0.0082 (7)	−0.0068 (7)
C9	0.0642 (12)	0.0715 (13)	0.0544 (11)	0.0027 (10)	0.0107 (10)	−0.0209 (10)
C10	0.0596 (12)	0.0847 (16)	0.0622 (11)	−0.0031 (11)	0.0254 (10)	0.0073 (11)
C11	0.0340 (8)	0.0451 (9)	0.0343 (7)	0.0036 (7)	0.0061 (6)	0.0065 (7)
C12	0.0404 (9)	0.0559 (10)	0.0414 (8)	0.0047 (8)	0.0104 (7)	−0.0042 (8)
C13	0.0339 (8)	0.0619 (11)	0.0490 (9)	0.0029 (8)	0.0122 (7)	−0.0034 (8)
C14	0.0351 (8)	0.0438 (9)	0.0439 (8)	0.0014 (7)	0.0038 (7)	0.0027 (7)
C15	0.0428 (9)	0.0504 (10)	0.0380 (8)	0.0055 (7)	0.0080 (7)	−0.0016 (7)
C16	0.0344 (8)	0.0541 (10)	0.0396 (8)	0.0058 (7)	0.0110 (6)	0.0046 (7)
C17	0.0519 (11)	0.0488 (11)	0.0774 (13)	0.0015 (8)	0.0105 (9)	−0.0121 (10)
C18	0.112 (2)	0.0563 (13)	0.0870 (16)	−0.0039 (13)	0.0250 (15)	0.0065 (12)
C19	0.0429 (9)	0.0578 (11)	0.0445 (9)	0.0004 (8)	0.0043 (7)	−0.0042 (8)
C20	0.0589 (11)	0.0538 (11)	0.0549 (10)	0.0054 (9)	0.0002 (9)	0.0030 (9)

### Geometric parameters (Å, °)

**Table:**

S1—O1	1.4256 (13)	C9—H9A	0.9600
S1—O2	1.4301 (12)	C9—H9B	0.9600
S1—N1	1.6458 (13)	C9—H9C	0.9600
S1—C11	1.7608 (16)	C10—H10A	0.9600
O3—C19	1.226 (2)	C10—H10B	0.9600
N1—C3	1.4534 (18)	C10—H10C	0.9600
N1—C1	1.480 (2)	C11—C12	1.386 (2)
N2—C19	1.352 (2)	C11—C16	1.386 (2)
N2—C14	1.436 (2)	C12—C13	1.379 (2)
N2—C17	1.470 (2)	C12—H12	0.9300
C1—C2	1.478 (3)	C13—C14	1.384 (2)
C1—H1A	0.9700	C13—H13	0.9300
C1—H1B	0.9700	C14—C15	1.385 (2)
C2—H2A	0.9600	C15—C16	1.383 (2)
C2—H2B	0.9600	C15—H15	0.9300
C2—H2C	0.9600	C16—H16	0.9300
C3—C8	1.376 (2)	C17—C18	1.494 (3)
C3—C4	1.383 (2)	C17—H17A	0.9700
C4—C5	1.390 (2)	C17—H17B	0.9700
C4—H4	0.9300	C18—H18A	0.9600
C5—C6	1.396 (2)	C18—H18B	0.9600
C5—C9	1.506 (2)	C18—H18C	0.9600
C6—C7	1.382 (2)	C19—C20	1.503 (3)
C6—C10	1.508 (2)	C20—H20A	0.9600
C7—C8	1.382 (2)	C20—H20B	0.9600
C7—H7	0.9300	C20—H20C	0.9600
C8—H8	0.9300		
			
O1—S1—O2	119.46 (8)	H9B—C9—H9C	109.5
O1—S1—N1	107.13 (7)	C6—C10—H10A	109.5
O2—S1—N1	107.03 (7)	C6—C10—H10B	109.5
O1—S1—C11	107.71 (8)	H10A—C10—H10B	109.5
O2—S1—C11	108.45 (7)	C6—C10—H10C	109.5
N1—S1—C11	106.37 (7)	H10A—C10—H10C	109.5
C3—N1—C1	116.91 (12)	H10B—C10—H10C	109.5
C3—N1—S1	115.41 (10)	C12—C11—C16	120.59 (15)
C1—N1—S1	116.17 (10)	C12—C11—S1	118.50 (12)
C19—N2—C14	123.41 (15)	C16—C11—S1	120.86 (12)
C19—N2—C17	119.09 (15)	C13—C12—C11	119.53 (15)
C14—N2—C17	117.48 (15)	C13—C12—H12	120.2
C2—C1—N1	111.47 (16)	C11—C12—H12	120.2
C2—C1—H1A	109.3	C12—C13—C14	120.17 (15)
N1—C1—H1A	109.3	C12—C13—H13	119.9
C2—C1—H1B	109.3	C14—C13—H13	119.9
N1—C1—H1B	109.3	C15—C14—C13	120.26 (15)
H1A—C1—H1B	108.0	C15—C14—N2	119.64 (15)
C1—C2—H2A	109.5	C13—C14—N2	120.09 (14)
C1—C2—H2B	109.5	C16—C15—C14	119.88 (15)
H2A—C2—H2B	109.5	C16—C15—H15	120.1
C1—C2—H2C	109.5	C14—C15—H15	120.1
H2A—C2—H2C	109.5	C15—C16—C11	119.56 (14)
H2B—C2—H2C	109.5	C15—C16—H16	120.2
C8—C3—C4	119.57 (14)	C11—C16—H16	120.2
C8—C3—N1	120.92 (13)	N2—C17—C18	113.53 (17)
C4—C3—N1	119.50 (13)	N2—C17—H17A	108.9
C3—C4—C5	121.45 (14)	C18—C17—H17A	108.9
C3—C4—H4	119.3	N2—C17—H17B	108.9
C5—C4—H4	119.3	C18—C17—H17B	108.9
C4—C5—C6	118.84 (15)	H17A—C17—H17B	107.7
C4—C5—C9	119.93 (16)	C17—C18—H18A	109.5
C6—C5—C9	121.23 (16)	C17—C18—H18B	109.5
C7—C6—C5	118.90 (15)	H18A—C18—H18B	109.5
C7—C6—C10	119.53 (17)	C17—C18—H18C	109.5
C5—C6—C10	121.57 (16)	H18A—C18—H18C	109.5
C8—C7—C6	121.90 (15)	H18B—C18—H18C	109.5
C8—C7—H7	119.0	O3—C19—N2	120.85 (18)
C6—C7—H7	119.0	O3—C19—C20	120.99 (18)
C3—C8—C7	119.31 (15)	N2—C19—C20	118.16 (16)
C3—C8—H8	120.3	C19—C20—H20A	109.5
C7—C8—H8	120.3	C19—C20—H20B	109.5
C5—C9—H9A	109.5	H20A—C20—H20B	109.5
C5—C9—H9B	109.5	C19—C20—H20C	109.5
H9A—C9—H9B	109.5	H20A—C20—H20C	109.5
C5—C9—H9C	109.5	H20B—C20—H20C	109.5
H9A—C9—H9C	109.5		
			
O1—S1—N1—C3	48.43 (12)	O2—S1—C11—C12	−159.96 (14)
O2—S1—N1—C3	177.67 (10)	N1—S1—C11—C12	85.22 (14)
C11—S1—N1—C3	−66.55 (12)	O1—S1—C11—C16	153.43 (13)
O1—S1—N1—C1	−169.22 (12)	O2—S1—C11—C16	22.83 (16)
O2—S1—N1—C1	−39.98 (14)	N1—S1—C11—C16	−91.99 (14)
C11—S1—N1—C1	75.80 (13)	C16—C11—C12—C13	0.2 (3)
C3—N1—C1—C2	−68.9 (2)	S1—C11—C12—C13	−177.06 (13)
S1—N1—C1—C2	149.37 (17)	C11—C12—C13—C14	−0.8 (3)
C1—N1—C3—C8	−39.7 (2)	C12—C13—C14—C15	0.9 (3)
S1—N1—C3—C8	102.32 (16)	C12—C13—C14—N2	180.00 (16)
C1—N1—C3—C4	140.40 (15)	C19—N2—C14—C15	−114.12 (19)
S1—N1—C3—C4	−77.54 (16)	C17—N2—C14—C15	67.4 (2)
C8—C3—C4—C5	−1.0 (2)	C19—N2—C14—C13	66.8 (2)
N1—C3—C4—C5	178.83 (13)	C17—N2—C14—C13	−111.71 (19)
C3—C4—C5—C6	1.4 (2)	C13—C14—C15—C16	−0.3 (3)
C3—C4—C5—C9	−178.33 (16)	N2—C14—C15—C16	−179.42 (15)
C4—C5—C6—C7	−0.7 (2)	C14—C15—C16—C11	−0.4 (3)
C9—C5—C6—C7	179.10 (17)	C12—C11—C16—C15	0.4 (2)
C4—C5—C6—C10	178.90 (16)	S1—C11—C16—C15	177.57 (13)
C9—C5—C6—C10	−1.3 (3)	C19—N2—C17—C18	−89.7 (2)
C5—C6—C7—C8	−0.5 (3)	C14—N2—C17—C18	88.9 (2)
C10—C6—C7—C8	179.92 (17)	C14—N2—C19—O3	−176.18 (16)
C4—C3—C8—C7	−0.2 (2)	C17—N2—C19—O3	2.3 (3)
N1—C3—C8—C7	179.99 (15)	C14—N2—C19—C20	4.7 (2)
C6—C7—C8—C3	0.9 (3)	C17—N2—C19—C20	−176.83 (17)
O1—S1—C11—C12	−29.36 (15)		

### Hydrogen-bond geometry (Å, °)

**Table:**

Cg1 is the centroid of the C3–C8 ring.

**Table:**

*D*—H···*A*	*D*—H	H···*A*	*D*···*A*	*D*—H···*A*
C8—H8···O1^i^	0.93	2.54	3.455 (2)	170
C10—H10a···Cg1^ii^	0.96	2.93	3.728 (2)	142

Symmetry codes: (i) −*x*+2, *y*−1/2, −*z*+1/2; (ii) −*x*+2, −*y*+1, −*z*+1.
